# *Kumbh Mela* Religious Gathering as a Massive Superspreading Event: Potential Culprit for the Exponential Surge of COVID-19 Cases in India

**DOI:** 10.4269/ajtmh.21-0601

**Published:** 2021-08-30

**Authors:** Ian Christopher N. Rocha, Mary Grace A. Pelayo, Sudhan Rackimuthu

**Affiliations:** ^1^ School of Medicine, Centro Escolar University, Manila, Philippines;; ^2^ Behavioral Sciences Department, De La Salle University, Manila, Philippines;; ^3^ Department of Medicine, Father Muller Medical College, Mangalore, Karnataka, India

## Abstract

The *Kumbh Mela* is a significant religious gathering of millions of Hindu devotees in India. It is celebrated on certain auspicious days in the Hindu calendar and attracts millions of pilgrims across the country. Despite the religious intention of millions of Hindu devotees, it raised public health concerns as it became a massive superspreading event for COVID-19. Being the second most populous country, India became the second most affected country during the COVID-19 pandemic. In addition to the arrival of severe acute respiratory syndrome coronavirus 2 (SARS-CoV-2) variants and the presence of the double mutated variant, which was first identified in India, the *Kumbh Mela* probably aggravated the country’s COVID-19 situation which resulted in an uncontrollable second wave. Several cases of COVID-19 across India had been contact-traced to returnees from the event who acted as a nidus to help spread the infection. As a consequence, India’s healthcare system was severely challenged as a result of the overwhelming hospitalizations and increasing fatalities resulting in an acute manpower shortage in healthcare along with the depletion of drugs and medical supplies despite being one of the largest pharmaceutical hubs globally. Leaders and governments around the world should learn from India’s experience and thereby take preventive measures to manage potential superspreading events to curb the spike of COVID-19 cases.

India, a predominantly Hindu country that occupies the greater part of South Asia, recently celebrated the *Kumbh Mela* or the “festival of pitcher,” a very significant religious gathering of millions of Hindu devotees and sages at the country’s riverbanks on April 1 to April 30, 2021, despite the threat of exponential surge of coronavirus disease 2019 (COVID-19) cases in the country.^1^^,^^2^ Being the second most populous country in the world with roughly one-sixth of the world’s population, India also became the second most affected country during the COVID-19 pandemic on April 12, 2021, while the country was in the middle of a month-long religious celebration.^3^^,^^4^ As of July 31, 2021, there are already 31,654,584 confirmed cases of COVID-19 in India, with 424,384 deaths.^5^

This grand religious gathering is usually celebrated on certain auspicious days in the Hindu calendar, which typically commences in January. However, because of the pandemic, the Indian government opted to postpone the event to April and shorten the festival duration to only 30 days from the usual, which is 100 days or more.^2^^,^^6^ It was also reported that weeks before the festival started, an impending second wave of COVID-19 cases was overtaking India and appeals to cancel the festival began circulating in the country. However, this was shut down by the government as the Prime Minister continued inviting devotees and assured them that it was clean and safe. As such, an overwhelming number of devotees, paying no visible observance to COVID-19 protocols, attended the religious gathering.^7^ Although the festival was postponed to a later date and shortened to 1 month, the *Kumbh Mela* still attracted millions of Hindu pilgrims across the country because of its religious significance and cultural traditions.

According to the Hindu scriptures, the gods and demons engaged in a 12-day fight for a pitcher containing the elixir of life. During the struggle, however, few drops of the elixir spilled on four places of the earth and these places became the venues for the *Kumbh Mela*. Since the fight ensued for 12 divine days, this is tantamount into 12 worldly years. Thus, four *Kumbh Mela* events are celebrated at four different places within a cycle of 12 years in India. These places are namely the Prayagraj in the state of Uttar Pradesh and Haridwar in the state of Uttarakhand both of which are situated on the banks of the river Ganges, along with Ujjain in Madhya Pradesh located on the bank of the river Shipra and at Nashik in Maharashtra positioned on the banks of the river Godavari.^1^

Despite the religious intention of millions of Hindu devotees, the *Kumbh Mela* raised major public health concerns as it likely became a massive superspreading event for the severe acute respiratory syndrome coronavirus 2 (SARS-CoV-2). The ritual practice of *Snan,* wherein the devout immerses oneself in the river water is undertaken, to attain *moksha* (salvation) as the water is believed to be transformed into a sacred elixir. Historically, the ritual was known to pose high concern to health as contamination of water can lead to public health outbreaks such as cholera, dysentery, typhoid, and other water-borne diseases.^1^ Despite its history of causing disease outbreaks, the *Kumbh Mela* was still celebrated for the whole month of April 2021 with an extremely large number of devotees amid the ongoing pandemic and continued spike of COVID-19 infections in the country. Preventive measures, such as practicing hand hygiene, wearing facemasks, and maintaining physical distance, were also grossly neglected by a majority of attendees.^3^^,^^7^ The threat of *Kumbh Mela* as a superspreader of SARS-CoV-2 was gravely apparent with the growing evidence of spread of infection linked back to the religious gathering.

The 1-month event was seen as a major culprit for the exponential surge of COVID-19 cases in the country. As depicted in Figure 1, the second wave of COVID-19 in India commenced during the massive superspreading event with an increasing incidence of cases. It can be observed that the daily new cases before the *Kumbh Mela* were not exceeding the 100,000-mark. However, a few days after its commencement, the daily new cases continuously escalated and eventually exceeded the 100,000-mark. In fact, even during the last day of the superspreading event, the daily new cases unfortunately reached the 400,000-mark for the first time, indicating the second wave’s peak. It can be gleaned that the highest peak, with 414,433 daily new cases, occurred a few days after the *Kumbh Mela*.^5^

**Figure 1.**
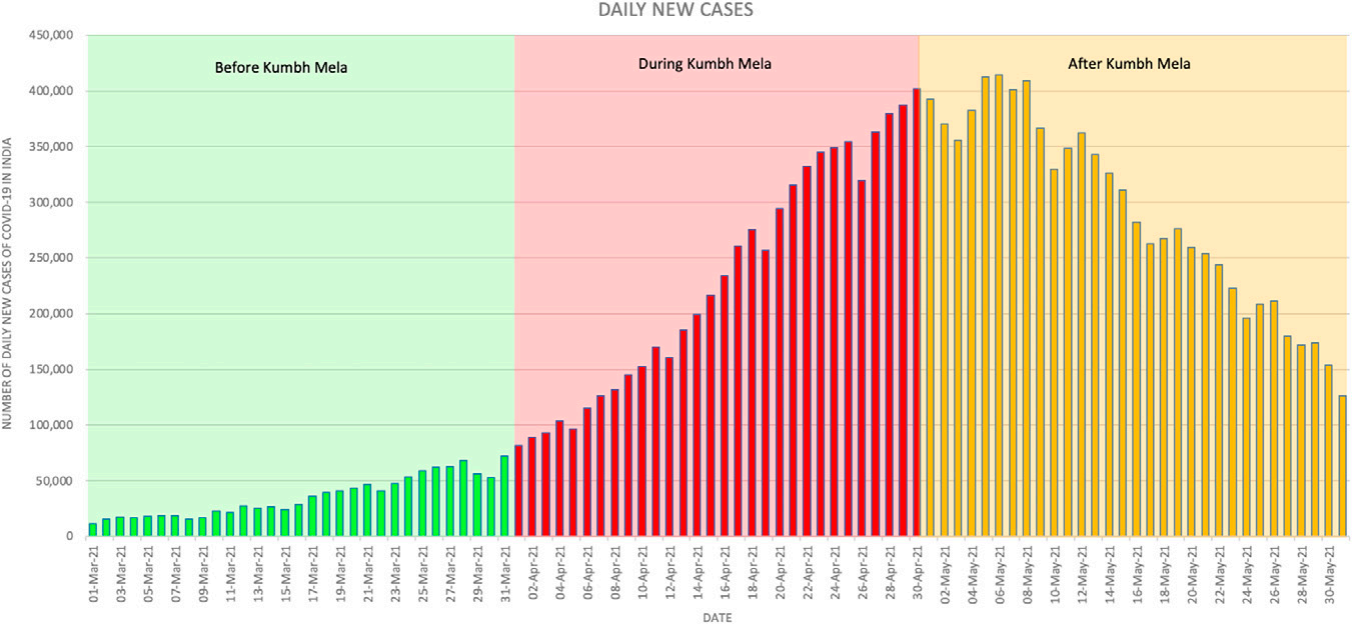
Daily new cases of COVID-19 in India before (March 1–31, 2021), during (April 1–30, 2021), and after (May 1–31, 2021) *Kumbh Mela*. This figure appears in color at www.ajtmh.org.

In addition to the recent massive superspreading event, the ongoing second wave of the COVID-19 pandemic was further exacerbated by the presence of the Delta variant, a double mutant variant of SARS-CoV-2, which was first identified in India and recently classified by the WHO as a variant of concern (VOC) because of its high transmissibility and global rapid spread.^8^^,^^9^ Additionally, the arrival of other highly transmissible SARS-CoV-2 VOCs in India, particularly the Alpha, Beta, and Gamma variants, may have also contributed in the surge of COVID-19 cases.^9^^,^^10^

As a result of the second wave of COVID-19, India’s healthcare system was greatly challenged because of the overwhelming hospitalizations and increasing fatalities throughout the country, resulting in a manpower shortage in healthcare and depletion of drugs and medical supplies.^4^^,^^11^^,^^12^ Patients with severe COVID-19 continued to suffocate because of shortage of oxygen supplies despite India being one of the largest pharmaceutical hubs in the world. The family members of the diseased as well as the public in India resorted to asking the government and private institutions for help in the hopes of obtaining oxygen and possible life-saving medications for those in need.^12^ In addition, the increasing mortality had also resulted in mass cremations of the dead in several makeshift areas as a result of the overwhelming number of deaths.^13^

Although the first wave of COVID-19 outbreak had been mitigated to an extent through movement restrictions and containment measures, the lockdown was subsequently lifted because of societal and economic pressures following the increased number of new cases leading to a devastating second wave. Despite the government’s efforts in reminding the people of continued observance of protective measures, such as hand hygiene, wearing face masks, observing social distancing, and imposing curfews, many citizens became complacent and exhibited irresponsible behaviors before and during the *Kumbh Mela* celebration. Many devotees traveled in crowded trains and other public transportation vehicles. There were also reported accounts of groups of mask-less pilgrims on the riverbanks while singing the glories of the Ganges. As stated by eminent epidemiologists, pilgrims presented an ideal setting for the virus to rapidly spread.^14^

Despite the grim state of the country before the onset of the event, there had been no significant talks or measures as to halt or limit the *Kumbh Mela* celebration, which became a cause for global concern. Critics attributed the reluctance of the Indian Prime Minister to cancel the mass gathering to the possible backlash from Hindu religious leaders since priests, seers, and ascetics play a significant role in gathering Hindu votes during elections. Uttarakhand’s former chief minister stated that he had initially planned to limit the celebration into a symbolic event as public health experts initially warned of new COVID-19 variants and the continued threat of the disease. However, this plan did not materialize as he was replaced by a new chief minister days before the festival who remarked that the blessings of *Ma Ganga* or the river goddess would protect them from the virus.^14^

As the second wave continues to ravage across India, several countries around the world have lent their help and support by sending out vaccines, drugs, oxygen concentrators, ventilators, and other necessary resources despite the shortage and increasing demand of medicines and hospital supplies globally.^12^^,^^15^ The Indian Prime Minister stated that oxygen will also be transported to hospitals from reserves that are under the military. An operation named “Co-jeet” was recently launched by the armed forces to aid the inoculation drive and to strengthen the anti-COVID-19 action plan by providing health-based resources to various states, which include transporting oxygen and providing medical assistance.^16^ The government has also been constantly pressing the public to get vaccinated because only 19% of its population received at least a single dose of COVID-19 vaccine and just 7% are fully inoculated as of July 31, 2021.^17^ Unlike other countries, India is still far from achieving herd immunity because of the slow vaccination rollout despite being the largest source of COVID-19 vaccines in the world.^12^^,^^17^^,^^18^ In addition, the government also urged its people to exercise extreme caution because of the surfeit of infections that continues to hit India. Complacency of the people has also further aggravated the problem at hand.

Public health experts stated that *Kumbh Mela* was possibly “the biggest superspreading event” in the pandemic history.^7^ Any large gathering in the future similar in nature to that of *Kumbh Mela* will most definitely still pose an important public health risk if the pandemic remains. Thus, prevention and mitigation of superspreading events will require quick recognition and understanding of the event that caused or may likely cause a wide transmission of the disease. After recognizing and understanding the dynamics of transmission, governments could then implement better control measures that would best mitigate the risk of widespread transmissions. Implementing public health interventions to prevent and mitigate superspreading events are equally crucial in the containment and mitigation phases.^19^^–^^21^ Therefore, we mention a few recommendations, as well as best practices from other countries, that the authorities can implement to hopefully curb the spread of the SARS-CoV-2, in events that might prove to be a potential superspreader.

Firstly, all participants must undergo mandatory reverse transcription polymerase chain reaction (RT-PCR) testing, the gold standard for diagnosing COVID-19, to ensure that all are not carriers of SARS-CoV-2. Secondly, the authorities must also limit the number of participants who will be attending the event, which would also help monitor the movement of people. Thirdly, the government, along with the other groups or organizations, should work closely to promote public health messages such as implementing protective measures throughout the juncture. Fourthly, leaders must also invest to ensure that adequate number of screening sites and handwashing stations are present throughout the area of gathering. Fifthly, vulnerable individuals including the elderly and the sick should be encouraged to not participate in person and in cases posing serious risk to the public, strict prohibition can be enforced. Lastly, emergency infrastructures for quarantining the infected must also be planned in preparation for untoward scenarios of overwhelming spread leading to increase in number of cases.

Some countries have successfully controlled superspreading events during the COVID-19 pandemic. Best examples were the temporary suspension of *Umrah* and *Hajj* pilgrimages in Saudi Arabia and the postponement of Olympic Games in Japan.^21^^–^^24^ In Saudi Arabia, the government provisionally suspended the *Umrah* pilgrimage, which was known to gather thousands of pilgrims daily, 2 days after detecting its first COVID-19 case in the country.^21^^,^^22^ The almost-three-month suspension provided them more time to plan on how to prevent the occurrence of superspreading event once the daily *Umrah* pilgrimage resumed and the annual *Hajj* pilgrimage commenced. The Saudi government only allowed pilgrims who were aged 20 to 65, healthy, not pregnant, nonreactive to COVID-19 through RT-PCR testing, and quarantined for 2 weeks before the pilgrimage. During their pilgrimage, the participants were assigned in safe “bubbles” with designated tracks and preventive measures. After their pilgrimage, participants underwent another RT-PCR test and quarantined for another 2 weeks before returning home.^22^ As a result of the successful mitigation plan of the Saudi government, there were no reported cases of COVID-19 during the pilgrimage.^23^

Furthermore, the Olympic Games in Japan, which was supposed to be held in 2020, was also postponed to 2021.^21^ Just like the pilgrimages in Saudi Arabia, the Japanese government also used the 1-year postponement to plan for better public health measures. The athletes were frequently subjected to RT-PCR testing, as well as quarantine, with the following sequence: two testing on 2 separate days within 3 days of their flight to Japan; another testing upon arrival in Japan; quarantine for 3 days; quarantine for 14 days if daily testing is refused; more daily testing upon arrival to the Olympic Village; more daily testing during the competition; and another testing after the Olympics and before leaving Japan.^24^ In addition, aside from the standard public health measures such as handwashing, social distancing, and avoiding crowded places, the other preventive protocols include immediate isolation of athletes who tested positive for COVID-19, strict prohibition of using public transportation and leaving safe “bubbles” among the athletes, and stern forbiddance of spectators to personally watch the games in the designated venues.^24^

The abovementioned recommendations and strategies can be emulated by other countries to prevent potential superspreading events such as what happened in India. Unfortunately, the case of *Kumbh Mela* as the likely culprit for the surge of COVID-19 cases in India’s second wave presented how complacency in observing COVID-19 preventive protocols and underestimating viral transmission have gone awry. Since this religious gathering depicts how societal and cultural underpinnings play a critical role in people’s behaviors, early engagement of communities and understanding people’s knowledge, attitudes, and practices are of great importance in designing emergency health responses specifically in the preventive and mitigation phases.^25^

It has been quite unfortunate that a large majority of people who attended *Kumbh Mela* to celebrate life have ironically faced the grim reality of having to face death themselves or among those around them. The COVID-19 situation in India is surely beyond heartbreaking. Societal beliefs and practices of people may at times be detrimental to themselves and others if observed without attention to public health concerns. As the global community suffers from the pandemic crisis, this article provides a perspective on how societies can help mitigate the further spread of the virus through observing COVID-19 preventive practices. Hence, everyone must remember to continue to remain vigilant with leaders and governments around the world hopefully learning from this ordeal, to prevent potential superspreading events from occurring in the future.
